# Activated Integrated Stress Response Induced by Salubrinal Promotes Cisplatin Resistance in Human Gastric Cancer Cells via Enhanced xCT Expression and Glutathione Biosynthesis

**DOI:** 10.3390/ijms19113389

**Published:** 2018-10-29

**Authors:** Sheng-Fan Wang, Chih-Hsuan Wung, Meng-Shian Chen, Chian-Feng Chen, Pen-Hui Yin, Tien-Shun Yeh, Yuh-Lih Chang, Yueh-Ching Chou, Hung-Hsu Hung, Hsin-Chen Lee

**Affiliations:** 1 Department of Pharmacy, Taipei Veterans General Hospital, Taipei 112, Taiwan; sfwang5@vghtpe.gov.tw (S.-F.W.); ylchang@vghtpe.gov.tw (Y.-L.C.); ycchou@vghtpe.gov.tw (Y.-C.C.); 2 Department and Institute of Pharmacology, School of Medicine, National Yang-Ming University, Taipei 112, Taiwan; knash031130@hotmail.com.tw (C.-H.W.); sp040755@gmail.com (M.-S.C.); 3 VYM Genome Research Center, National Yang-Ming University, Taipei 112, Taiwan; cfchen@ym.edu.tw; 4 Department of Medical Research, Taipei Veterans General Hospital, Taipei 112, Taiwan; phyin@vghtpe.gov.tw; 5 Institute of Anatomy and Cell Biology, School of Medicine, National Yang-Ming University, Taipei 112, Taiwan; tsyeh@ym.edu.tw; 6 School of Pharmacy, Taipei Medical University, Taipei 110, Taiwan; 7 Division of Gastroenterology, Department of Medicine, Cheng Hsin General Hospital, Taipei 112, Taiwan; 8 Faculty of Medicine, School of Medicine, Institute of Clinical Medicine and Genomic Research Center, National Yang-Ming University, Taipei 112, Taiwan

**Keywords:** gastric cancer, integrated stress response (ISR), cisplatin resistance, salubrinal, xCT

## Abstract

The integrated stress response (ISR) pathway is essential for adaption of various stresses and is related to mitochondrion-to-nucleus communication. Mitochondrial dysfunction-induced reactive oxygen species (ROS) was demonstrated to activate general control nonderepressible 2 (GCN2)–eukaryotic translation initiation factor 2α (eIF2α)–activating transcription factor-4 (ATF4) pathway-mediated cisplatin resistance of human gastric cancer cells. However, whether or how ISR activation per se could enhance chemoresistance remains unclear. In this study, we used eIF2α phosphatase inhibitor salubrinal to activate the ISR pathway and found that salubrinal reduced susceptibility to cisplatin. Moreover, salubrinal up-regulated ATF4-modulated gene expression, and knockdown of ATF4 attenuated salubrinal-induced drug resistance, suggesting that ATF4-modulated genes contribute to the process. The ATF4-modulated genes, xCT (a cystine/glutamate anti-transporter), tribbles-related protein 3 (TRB3), heme oxygenase 1 (HO-1), and phosphoenolpyruvate carboxykinase 2 (PCK2), were associated with a poorer prognosis for gastric cancer patients. By silencing individual genes, we found that xCT, but not TRB3, HO-1, or PCK2, is responsible for salubrinal-induced cisplatin resistance. In addition, salubrinal increased intracellular glutathione (GSH) and decreased cisplatin-induced lipid peroxidation. Salubrinal-induced cisplatin resistance was attenuated by inhibition of xCT and GSH biosynthesis. In conclusion, our results suggest that ISR activation by salubrinal up-regulates ATF4-modulated gene expression, increases GSH synthesis, and decreases cisplatin-induced oxidative damage, which contribute to cisplatin resistance in gastric cancer cells.

## 1. Introduction

Although the incidence of gastric cancer has declined and reached a plateau, and although gastric cancer mortality is declining due to improvements in surgical and systemic treatments as well as screening, early detection, and treatment strategies for *Helicobacter pylori* infection, gastric cancer is still a considerable global health burden [1]. Surgery is the major treatment for patients with local gastric cancer. For patients with metastatic disease, systemic chemotherapy is the most effective treatment modality and could adequately palliate the symptoms of gastric cancer [2]. The 5-Fluorouracil (5-FU) derivative and platinum medications are often prescribed for systemic chemotherapy to treat gastric cancer [3,4,5]. Despite the acceptable efficacy of systemic combination chemotherapy treatment, some gastric cancer patients relapsed after several months of treatment [6]. Hence, chemotherapy resistance-mediated cancer progression is still an important issue for the treatment of gastric cancer patients.

Over the last 50 years, a number of platinum analogues had been discovered to expand the spectrum of anti-tumor activity and/or reduce the toxicity of first (e.g., cisplatin) and second/third generation (e.g., carboplatin and oxaliplatin) platinum drugs [7]. Cisplatin had been widely used in various cancers and in widespread clinical use for more than a generation. Cisplatin is widely used for adjuvant chemotherapy in early-stage gastric cancer patients and systemic/palliative chemotherapy in advanced-stage gastric cancer patients. Cisplatin is a platinum containing agent and is hydrated to form a positively charged species, and could interact with DNA of cancer cells. Cisplatin has been characterized as a DNA linkage agent, and the cytotoxicity of cisplatin has generally contributed to the ability to form intra-strand and inter-strand DNA linkage [8]. Cisplatin is highly toxic for proliferating cancer cells, due to it forming adducts with DNA and impeding DNA replication and mitosis [9]. Exposure of cancer cells to cisplatin may cause mitochondrial alterations leading to activation of apoptosis or cell death [10]. In addition, cisplatin can induce oxidative and reticular stress. Although cisplatin was reported to induced DNA-adduct lesions in the nuclear regions and mitochondrial DNA (mtDNA) was disproportionately less affected [11], some lines of evidence showed that cisplatin bind to mtDNA with higher efficiency than to nuclear DNA [12,13].

Cisplatin resistance has been investigated for several years, and at least four aspects about cisplatin resistance have been proposed (pre-, on-, post-, and off-target) [14]. In the pre-target aspect, there were several transporters that were identified as associated with cisplatin resistance, such as copper transporter 1 (CTR1), copper-transporting ATPase (ATP7B), multidrug resistance-associated protein 2 (MRP2), and volume-regulated anion channels (VRACs) [15,16,17,18]. The increased repair system for the molecular damage caused by cisplatin, such as excision repair cross-complementing rodent repair deficiency, complementation group 1 (ERCC1), might be involved in on-target resistance [19]. To diminish the signal transduction of cisplatin-induced cell senescence or apoptosis and to increase pro-survival, cellular signals might contribute to post-target and off-target resistance, such as bcl-2 family members and the akt pathway [20,21,22].

Integrated stress response (ISR) is a mechanism by which mammalian cells adapt to intrinsic cellular stress (such as endoplasmic reticulum stress or haemoglobin deficiency) and extrinsic cellular stress (such as nutrient deficiency, viral infection, or hypoxia) through the regulation of amino acid transporters, antioxidant response, and chaperones [23,24,25]. Under stress conditions, the eukaryotic translation initiation factor 2α (eIF2α) is phosphorylated by eIF2 kinases and inhibits cap-dependent protein translation. On the other hand, the phosphorylation of eIF2α transmits the stress response through the up-regulation of the activating transcription factor-4 (ATF4) [25]. Four eIF2 kinases have been identified to be responsible for eIF2α phosphorylation, such as protein kinase R (PKR)-like endoplasmic reticulum kinase (PERK, responsible for endoplasmic reticulum stress), general control nonderepressible 2 (GCN2, activated by amino acid starvation), protein kinase R (PKR, up-regulated by viral infections), and heme-regulated eIF2α kinase (HRI, induced by oxidative stress or heme deprivation) [26,27,28,29]. The eIF2α–ATF4 pathway not only maintains the cellular redox homeostasis, but also regulates cellular metabolism and nutrient uptake [30,31]. This pathway is also important for the adaptation of tumour cells to hypoxic stress and contributes to tumour growth [32] as well as chemotherapy resistance [33,34,35,36].

PERK and GCN2 were suggested to contribute to the activation of the ISR in response to mitochondrial stress [37,38,39]. Recently, mitochondrial dysfunction-induced reactive oxygen species (ROS) production was demonstrated to activate the GCN2–eIF2α–ATF4 pathway and induce cisplatin resistance in human gastric cancer cells [40]. However, it is unclear whether the activation of ISR per se is sufficient for the development of drug resistance. In the present study, we used the eIF2α phosphatase inhibitor salubrinal [41] to persistently activate the eIF2α–ATF4 pathway and evaluated the role of ISR in the cisplatin resistance of human gastric cancer cells. The ATF4-regulated genes were further evaluated to understand their role in the mechanism of cisplatin resistance.

## 2. Results

### 2.1. Salubrinal Activates ISR and Induces Cisplatin Resistance in Gastric Cancer Cells

**Figure 1:**
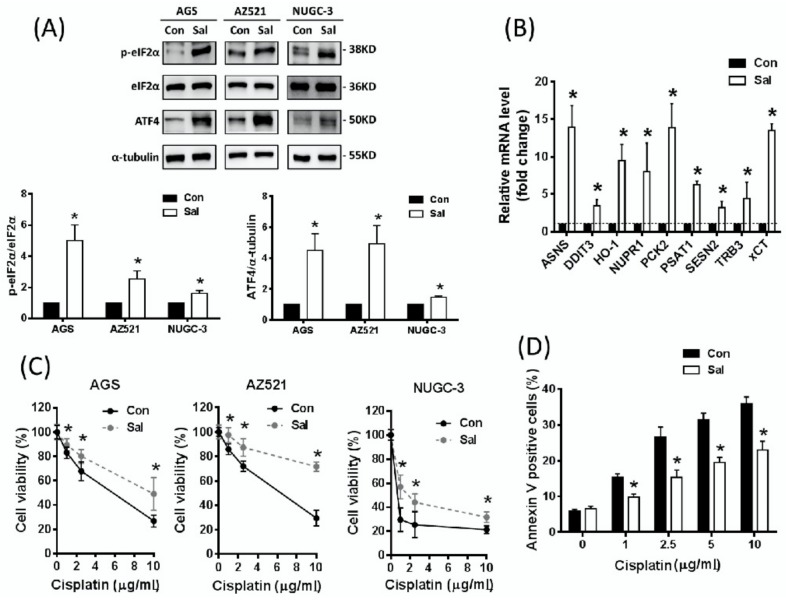
Salubrinal activates integrated stress response (ISR) and induces cisplatin resistance in gastric cancer cells. (**A**) The AGS, AZ521, and NUGC-3 gastric cancer cells were treated with salubrinal (Sal, 30 µM) for 24 h and the eukaryotic translation initiation factor 2α (eIF2α)–activating transcription factor-4 (ATF4) pathway (ISR) was analysed by Western blot analysis. The immunoblot values were normalized to α-tubulin. (**B**) The AGS gastric cancer cells were treated with 30 µM Sal for 24 h. The ATF4-regulated gene expression was determined using quantitative real-time reverse transcription-polymerase chain reaction (q-RT PCR). (**C**) The AGS, AZ521, and NUGC-3 gastric cancer cells were pre-treated with 30 µM Sal for 24 h, followed by co-treatment with cisplatin for 48 h. The cell viability was determined by the Sulforhodamine B (SRB) assay. (**D**) The Sal-induced cisplatin resistance was further validated by determination of the annexin V-positive population. AGS cells were pretreated with 30 µM Sal for 24 h and then co-treated with 30 µM Sal and cisplatin for 48 h. Data represent the mean ± standard error of mean SEM (standard error of the mean) of three independent experiments. *: *p* < 0.05, compared with the control cells or non-salubrinal pretreated cells.

To evaluate whether the activation of ISR is able to induce cisplatin resistance, we treated three human gastric cancer cell lines (AGS, AZ521, NUGC-3) with the eIF2α phosphatase inhibitor salubrinal to maintain the phosphorylation status of eIF2α and to increase ATF4 protein expression levels (Figure 1A). Moreover, the increased transcription activity of ATF4 was demonstrated by quantitative real-time reverse transcription-polymerase chain reaction (q-RT PCR) analysing the ATF4-associated gene [42]. The expression of several ATF4-regulated genes is significantly up-regulated by salubrinal treatment (Figure 1B). These results indicate that salubrinal may activate the ISR pathway in these gastric cancer cells. Importantly, we found that salubrinal significantly decreases cellular sensitivity to cisplatin (Figure 1C) and decreases the cisplatin-induced annexin V-positive population (Figure 1D). These results suggest that salubrinal activates ISR and induces cisplatin resistance in gastric cancer cells.

### 2.2. ATF4 Plays an Essential Role in Salubrinal-Induced Cisplatin Resistance in Gastric Cancer Cells

**Figure 2:**
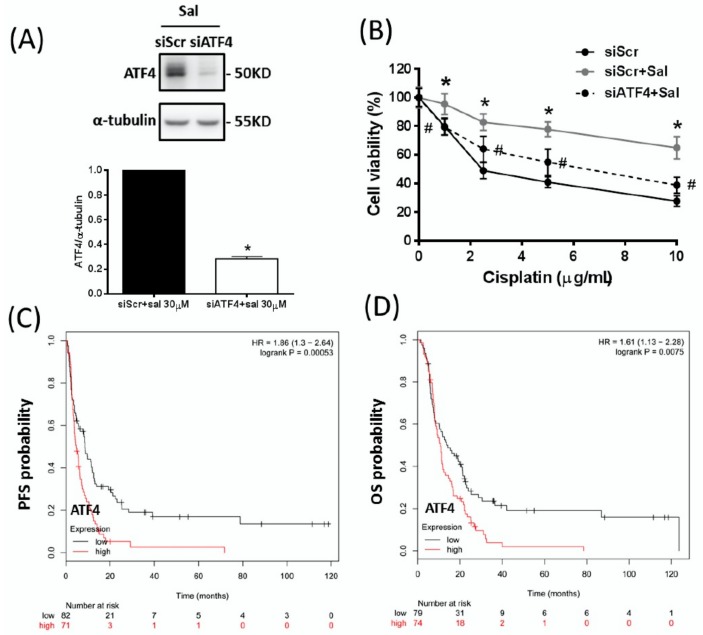
ATF4 plays an essential role in salubrinal-induced cisplatin resistance in gastric cancers cells. (**A**) Specific small interfering (si)RNA against ATF4 (150 pmol for 2 × 10^5^ cells in a six-well plate) was used to knockdown ATF4 in AGS gastric cancer cells, and the knockdown efficiency was determined by Western blotting. The control cells with scrambled siRNA (siScr) and the ATF4-knockdown cells (siATF4) were treated with 30 µM salubrinal (Sal) for 24 h. (**B**) The control cells with scrambled siRNA and the ATF4-knockdown cells were pre-treated with 30 µM salubrinal for 24 h and then co-treated with 30 µM Sal and cisplatin for 48 h. The cell viability was analysed by the SRB assay. (**C**,**D**) Kaplan–Meier survival analyses performed using the Kaplan–Meier plotter online database (Available online: http://kmplot.com/analysis/) showed the effect of ATF4 expression on (**C**) progression-free survival (PFS) and (**D**) overall survival (OS) in the subgroup of gastric cancer patients (5-Fluorouracil (5-FU)-based adjuvant treatments). Data are shown as the mean ± SEM of three independent experiments; * *p* < 0.05, compared with the siScr control group. ^#^ *p* < 0.05, compared with salubrinal-treated siScr cells.

To verify the essential role of ATF4 in salubrinal-induced cisplatin resistance, we used specific small interfering (si)RNA to knockdown the expression of ATF4 in the AGS gastric cancer cells. We found that the knockdown of ATF4 can attenuate the salubrinal-up-regulated ATF4 expression and cisplatin resistance (Figure 2A,B). Moreover, we found that patients with higher ATF4 expression in gastric cancer under adjuvant chemotherapy treatment have a lower progression-free survival (PFS) (hazard ratio (HR): 1.86, 1.3–2.64, log rank *p* = 0.00053, Figure 2C) and a lower overall survival (OS) (HR: 1.61, 1.13–2.28, log rank *p* = 0.0075, Figure 2D) than patients with lower ATF4 expression, suggesting that higher ATF4 expression is a poorer prognostic factor for gastric cancer patients who received adjuvant chemotherapy treatment. These results reveal that ATF4 plays a critical role in the salubrinal-induced cisplatin resistance.

### 2.3. Some of the ATF4-Regulated Genes, TRB3, HO-1, PCK2, and xCT, are Associated with Gastric Cancer Patients with Poor Prognosis after Adjuvant Chemotherapy

To further identify the ATF4-regulated genes that are responsible for cisplatin resistance, we used q-RT PCR to verify the microarray results (Supplementray Table S1) and analyzed them using the ingenuity pathway analysis (IPA). The IPA shows that the salubrinal-up-regulated genes tribbles-related protein 3 (TRB3), heme oxygenase 1 (HO-1), phosphoenolpyruvate carboxykinase (PCK2), and xCT might be involved in tumour cell survival, cell proliferation, and cell death (Supplementray Table S2). In addition, we found that patients with higher TRB3, HO-1, PCK2, and xCT expression in gastric cancer under adjuvant chemotherapy treatment have lower PFS and lower OS than patients with lower TRB3, HO-1, PCK2, and xCT expression, respectively (Table 1).

**Figure 3:**
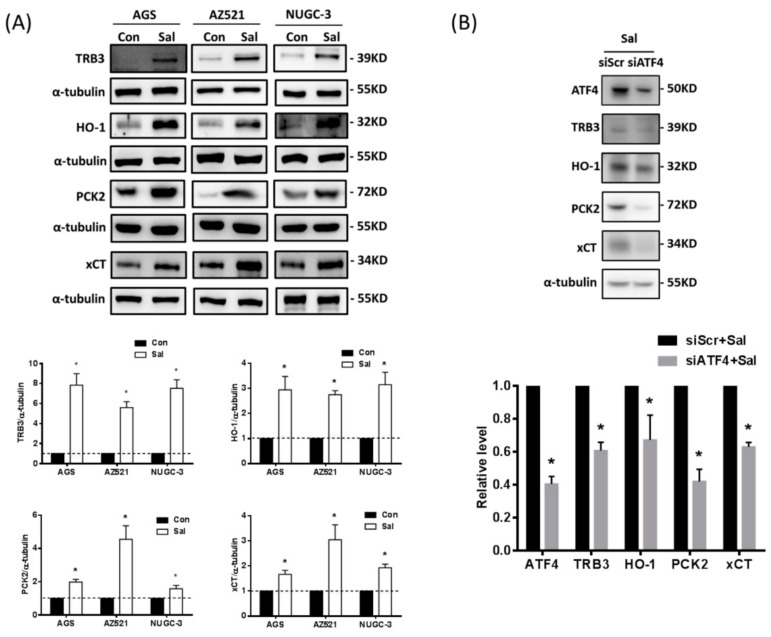
Salubrinal-up-regulated expression of TRB3, HO-1, PCK2 and xCT is ATF4-dependent. (**A**) The AGS, AZ521, and NUGC-3 gastric cancer cells were treated with salubrinal (Sal, 30 µM) for 24 h and the expression of tribbles-related protein 3 (TRB3), heme oxygenase 1 (HO-1), phosphoenolpyruvate carboxykinase (PCK2), and xCT was analysed by Western blot analysis. (**B**) Specific siRNA against ATF4 (150 pmol for 2 × 10^5^ cells in a six-well plate) was used to knockdown ATF4 in the AGS gastric cancer cells, and the knockdown efficiency and the changes in TRB3, HO-1, PCK2, and xCT were determined by Western blotting. The control cells with scrambled siRNA (siScr) and the ATF4-knockdown cells (siATF4) were also treated with 30 µM Sal for 24 h. The immunoblot values were normalized to α-tubulin. Data are shown as the mean ± SEM of three independent experiments; * *p* < 0.05, compared with the control group of siScr cells.

Using Western blotting analysis, we verified that the protein expression of these four genes is significantly up-regulated by salubrinal (Figure 3A). To confirm that the up-regulation of these four genes is ATF4-dependent, we used siRNAs to knock down ATF4 and found that the salubrinal-up-regulated expression of these four proteins is significantly attenuated (Figure 3B). These results indicate that the salubrinal-up-regulated expression of TRB3, HO-1, PCK2, and xCT is ATF4-dependent. These results suggest that TRB3, HO-1, PCK2, and xCT have the potential to be involved in the ISR-enhanced cisplatin resistance.

### 2.4. Up-Regulated Expression of xCT, but not of TRB3, HO-1, or PCK2, Contributes to the Salubrinal-Induced Cisplatin Resistance

**Figure 4:**
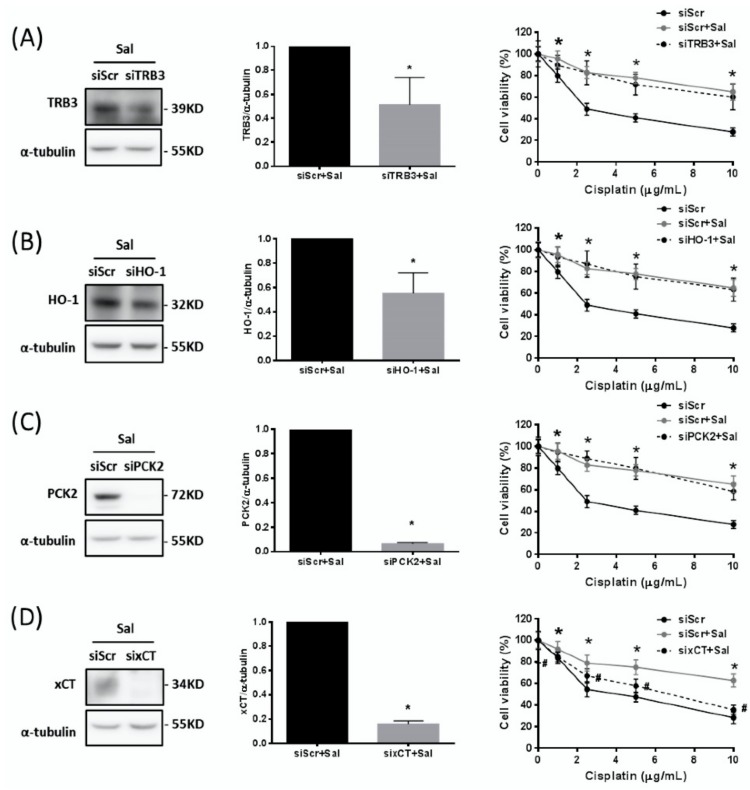
Up-regulated expression of xCT, but not of TRB3, HO-1, or PCK2, contributes to the salubrinal-induced cisplatin resistance. (**A**–**D**) Specific siRNAs against (**A**) TRB3, (**B**) HO-1, (**C**) PCK2, and (**D**) xCT (150 pmol for 2 × 10^5^ cells in a six-well plate) were used to knock down TRB3, HO-1, PCK2, and xCT, respectively, in AGS gastric cancer cells, and the knockdown efficiency was determined using Western blotting. The control cells with scrambled siRNA (siScr) and the TRB3-, HO-1-, PCK2-, and xCT-knockdown cells (siTRB3, siHO-1, siPCK2, and sixCT) were treated with 30 µM salubrinal (Sal) for 24 h. The cell viability was analysed by the SRB assay. The siScr cells and the siTRB3, siHO-1, siPCK2, and sixCT cells were pretreated with 30 µM Sal for 24 h, and then co-treated with 30 µM Sal and cisplatin for 48 h. Data are shown as the mean ± SEM of three independent experiments; * *p* < 0.05, compared with the siScr control group; *^#^ p* < 0.05, compared with salubrinal-treated siScr cells.

To examine whether the up-regulation in expression of TRB3, HO-1, PCK2, or xCT by salubrinal contributes to cisplatin resistance, we used specific siRNA to knock down the expression of TRB3, HO-1, PCK2, and xCT. We found that knockdown of the expression of TRB3 (Figure 4A), HO-1 (Figure 4B), and PCK2 (Figure 4C) could not attenuate the salubrinal-induced cisplatin resistance. Conversely, knockdown of xCT expression significantly repressed the salubrinal-induced cisplatin resistance (Figure 4D). These results indicate that the up-regulation of expression of xCT, but not of TRB3, HO-1, or PCK2, might contribute to the salubrinal-induced cisplatin resistance.

### 2.5. Salubrinal-Induced xCT Expression is Associated with Increased Intracellular Glutathione (GSH) Biosynthesis and Decreased Cisplatin-Induced Oxidative Stress

**Figure 5:**
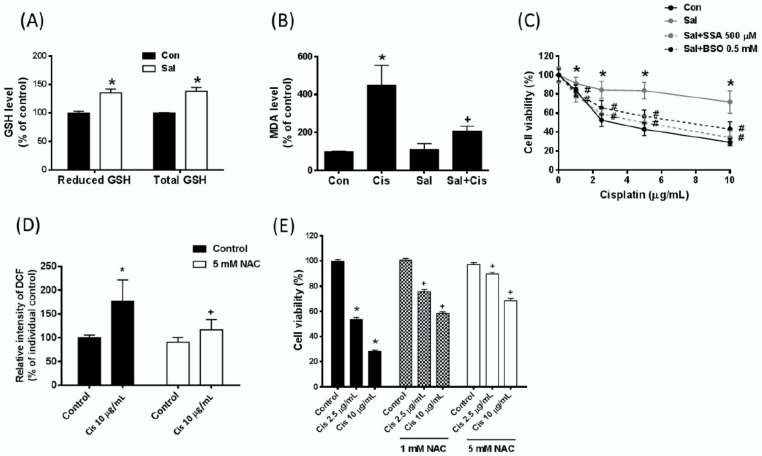
Salubrinal-induced xCT expression is associated with increased intracellular glutathione (GSH) biosynthesis and decreased cisplatin-induced oxidative stress. (**A**) The AGS cells were treated with salubrinal (Sal, 30 µM) for 24 h. After treatment, the (6 × 10^5^) cells were collected, and the total/reduced GSH levels were analysed by the GSH assay kit. (**B**) The AGS cells were pre-treated with 30 µM Sal for 24 h and then co-treated with 30 µM Sal and cisplatin (10 µg/mL) for 48 h. The level of lipid peroxidation was determined by the malondialdehyde (MDA) assay kit. (**C**) The AGS gastric cancer cells were pre-treated with 30 µM Sal for 24 h and then co-treated with 30 µM Sal, cisplatin, sulfasalazine (SSA), and buthionine sulfoximine (BSO) for 48 h. The cell viability was analysed by the SRB assay. (**D**) The AGS cells were pre-treated with 5 mM N-acetyl cysteine (NAC) for 24 h, followed by co-treatment with cisplatin (Cis, µg/mL) for 24 h. The cellular ROS level was determined by flow cytometry with dichlorodihydrofluorescein (DCF) staining. (**E**) The AGS cells were pre-treated with 1 and 5 mM NAC for 24 h, followed by co-treatment with cisplatin (Cis) for 48 h. The cell viability was determined by the SRB assay. Data are shown as the mean ± SEM of three independent experiments; * *p* < 0.05, compared with the control group. ^#^ *p* < 0.05, compared with salubrinal-treated cells, + *p* < 0.05, compared with cisplatin-treated AGS cells or individual non-NAC-pretreated AGS parental cells.

Based on the function of xCT as a cystine/glutamate anti-transporter, up-regulation of xCT expression might increase cystine uptake from the extracellular environment and enhance GSH biosynthesis. We thus examined whether salubrinal treatment could increase the intracellular GSH level. The results reveal that salubrinal treatment increases intracellular levels of both the reduced form and total GSH (Figure 5A). To evaluate whether salubrinal treatment could reduce the cisplatin-induced oxidative stress, we analysed the effect of salubrinal on the cisplatin-induced increase in lipid peroxidation malondialdehyde (MDA) levels. We found that salubrinal significantly attenuates the cisplatin-induced increase in lipid peroxidation (Figure 5B). Moreover, we found that sulfasalazine (SSA, xCT inhibitor) and buthionine sulfoximine (BSO, GSH synthesis inhibitor) significantly attenuate the salubrinal-induced cisplatin resistance (Figure 5C). These results suggest that the up-regulation of both xCT and GSH biosynthesis contribute to the salubrinal-induced cisplatin resistance.

In addition, we treated the gastric cancer cells with the GSH precursor N-acetylcysteine (NAC) combined with cisplatin and found that NAC significantly reduces the cisplatin-induced ROS levels (Figure 5D) and attenuates cisplatin-induced cell death (Figure 5E). These results suggest that increased xCT expression may contribute to the salubrinal-induced cisplatin resistance through enhanced GSH biosynthesis and reduced cisplatin-induced oxidative stress.

### 2.6. High xCT Expression Contributes to Cisplatin Resistance of the Cisplatin-Resistant Gastric Cancer Cells

**Figure 6:**
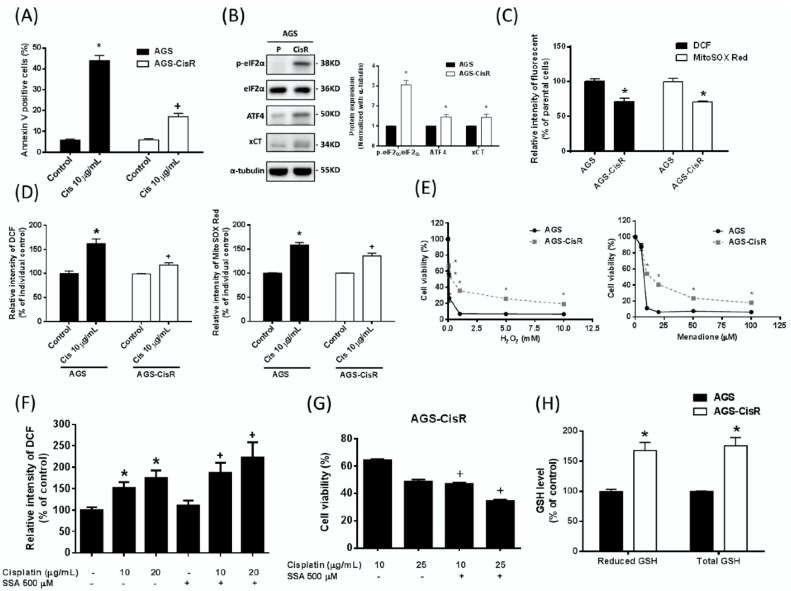
High expression of xCT contributes to cisplatin resistance in gastric cancer cells. (**A**) The chemoresistance in the cisplatin-resistant gastric cancer cells was further validated by the apoptosis assay. The AGS and AGS–CisR gastric cancer cells were seeded at a density of 2 × 10^5^ cells per well in six-well plates and were cultured with cisplatin (Cis) for 48 h. (**B**) The integrated stress response eIF2α–ATF4–xCT pathway between the parental and the cisplatin-resistant gastric cancer cells was evaluated by eIF2α phosphorylation and by ATF4 and xCT induction. The immunoblot values were normalized to α-tubulin. (**C**) The AGS and AGS–CisR gastric cancer cells were seeded at a density of 3 × 10^5^ cells per well in six-well plates and were cultured overnight before the determination of the basal cellular ROS level and the mitochondrial ROS level. (**D**) The AGS and AGS–CisR cells were seeded at a density of 2 × 10^5^ cells per well in six-well plates and were cultured with cisplatin (Cis) for 24 h. The cellular ROS level and mitochondrial ROS level were determined by flow cytometry with DCF and MitoSOX Red staining, respectively. (**E**) The AGS and AGS–CisR cells were treated with hydrogen peroxide (H_2_O_2_) and menadione for 48 h. The cell viability was determined by the SRB assay. (**F**) The AGS–CisR cells were seeded at a density of 2 × 10^5^ cells per well in six-well plates and were cultured with cisplatin and/or sulfasalazine (SSA) for 24 h. The cellular ROS level was determined by flow cytometry with DCF staining. (**G**) The AGS–CisR cells were treated with 500 µM SSA and cisplatin for 48 h. The cell viability was determined by the SRB assay. (**H**) The AGS and AGS-CisR cells were seeded in a 6-cm dish at a density of 3 × 10^5^ cells and were cultured overnight prior to determination. The total/reduced GSH levels were analysed by the GSH assay kit. Data represent the mean ± SEM of three independent experiments. * *p* < 0.05, compared with the parental cells or control group; + *p* < 0.05, compared with the cisplatin-treated AGS parental cells or individual non-SSA cotreated AGS–CisR cells.

To further verify the role of xCT in cisplatin resistance, we established cisplatin-resistant gastric cancer cell lines by increasing the exposure concentration of cisplatin until cells could tolerate the IC_50_ of cisplatin for six months, as in a previous study [40]. The cisplatin-resistant gastric cancer cells exhibit higher cell viability against cisplatin than parental cells (half maximal inhibitory concentration IC_50Cisplatin_: 2.31 µg/mL for AGS, 15.83 µg/mL for AGS-CisR). Under 10 µg/mL cisplatin treatment, the apoptosis levels of the cisplatin-resistant cancer AGS–CisR cells are significantly lower than those of the parental AGS cells (Figure 6A). Moreover, the phosphorylation levels of eIF2α and the protein expression of ATF4 and xCT of the cisplatin-resistant AGS–CisR cancer cells were higher than those of the parental AGS cells (Figure 6B). These results indicate that the ISR–xCT pathway is elevated in the cisplatin-resistant gastric cancer cells. To evaluate whether the increased antioxidant ability could contribute to the cisplatin resistance of the cisplatin-resistant gastric cancer cells, we analysed intracellular ROS changes in response to cisplatin treatment. We found that both the basal cellular ROS and mitochondrial ROS levels of the cisplatin-resistant AGS–CisR cells are significantly lower than those of the parental AGS cells (Figure 6C). Moreover, under 10 µg/mL cisplatin treatment, both the basal cellular ROS and mitochondrial ROS levels of the cisplatin-resistant AGS–CisR cells are significantly lower than those of the parental AGS cells (Figure 6D). In addition, the AGS–CisR cells were more resistant to H_2_O_2_ (hydrogen peroxide) and menadione (Figure 6E). We further found that inhibition of xCT by SSA elevates the cisplatin-induced ROS production (Figure 6F) and attenuates the cisplatin resistance of the AGS–CisR cells (Figure 6G). Moreover, the intracellular levels of both the reduced form and total GSH are elevated in the AGS–CisR cells (Figure 6H). These results indicate that the activation of ISR and the high expression of xCT-mediated antioxidant ability may contribute to the cisplatin resistance of gastric cancer cells.

## 3. Discussion

In this study, we used salubrinal (an eIF2α phosphatase inhibitor) to demonstrate that activation of the eIF2α–ATF4 pathway itself may enhance the cisplatin resistance of human gastric cancer cells. Using siRNA to knock down the expression of ATF4, we found that the salubrinal-induced cisplatin resistance is ATF4-dependent. Moreover, higher expression levels of ATF4 and its downstream genes TRB3, HO-1, PCK2, and xCT in gastric cancers were significantly correlated with poorer prognosis for patients receiving chemotherapy. These findings suggest the essential role of the activation of the eIF2α–ATF4 pathway in drug resistance of gastric cancer.

TRB3 is one of the mammalian homologue Tribbles isoforms that are serine/threonine kinases lacking catalytic activity [43]. Tribbles can interact with different proteins and regulate different biological functions, including functions involved in diabetes, stress-response, and development [43]. In addition, TRB3 could regulate the cell cycle, suppress cell survival, affect DNA repair, and maintain genome stability, and it is overexpressed in various cancer cells [44,45]. The tribbles family was thus thought to play an important role in cancer development and progression [46]. TRB3 was found to be induced by the ATF4–C/EBP homologous protein (CHOP) pathway and to be responsible for endoplasmic reticulum stress-dependent cell death [47]. Although TRB3 is related to cell death and salubrinal treatment can activate TRB3 expression, knockdown of TRB3 does not affect the salubrinal-induced cisplatin resistance in the present study. Therefore, TRB3 may not be involved in the development of ISR-mediated cisplatin resistance in gastric cancers.

HO-1 is a stress-inducible enzyme, which is a rate-limiting enzyme catalysing the oxidative degradation of cellular heme to free iron, carbon monoxide (CO), and biliverdin [48]. HO-1 has antioxidant and anti-inflammatory abilities via biliverdin and CO. Recently, CO was found to contribute to the chemoresistance and adaption of oxidative stress in cancer cells by inhibiting the heme-containing cystathionine β-synthase, and was found to reprogram glucose metabolism to the pentose phosphate pathway, resulting in a subsequent increase in nicotinamide adenine dinucleotide phosphate (NADPH) and a replenishment of reduced GSH [49]. HO-1 was found to be extensively expressed in various human cancers, where it serves as an important regulator of survival by modulating apoptosis and angiogenesis [50]. Moreover, the expression level of HO-1 was found to be positively correlated with disease stage and poor prognosis in patients [51]. HO-1 could protect cancer cells from chemotherapy- or radiation therapy-induced apoptosis, suggesting that it may be involved in cancer treatment resistance [52,53]. Even through HO-1 is related to chemoresistance and salubrinal treatment can activate HO-1 expression, knockdown of HO-1 does not affect the salubrinal-induced cisplatin resistance in our study. The results suggest that HO-1 is not involved in the ISR-mediated cisplatin resistance in gastric cancers and the increment of GSH by ISR may not be through HO-1 induction. The biological function of salubrinal-elevated HO-1 expression should be further investigated.

PCK2 is a mitochondrial isoform of the phosphoenolpyruvate carboxykinase (PEPCK) that catalyses the conversion of oxaloacetate to phosphoenolpyruvate in the presence of guanosine triphosphate. PEPCK is the rate-limiting step in the metabolic pathway that produces glucose from lactate and other precursors derived from the citric acid cycle. PEPCK and PCK2 enabled phosphoenolpyruvate conversion from non-carbohydrate substrates to support tumour growth [54]. In a previous study, PCK2 activation was found to be important for the response to environmental stresses such as glucose depletion, conferring an adaptive ability on cancer cells [55,56]. Furthermore, chemotherapy (such as gemcitabine) and mitochondrial inhibitors (FCCP) could increase the gene expression of PCK2 [35,57]. PCK2 can provide several intermediate sources for cell growth such as NADPH. NADPH provides reducing materials for the biosynthetic reactions and oxidation-reduction reactions that are involved in protecting against the toxicity of ROS and in allowing the regeneration of GSH. Despite the increase in PCK2 expression following salubrinal treatment, knockdown of PCK2 does not affect the salubrinal-induced cisplatin resistance. Therefore, PCK2 is not involved in the ISR-mediated cisplatin resistance in gastric cancers and NADPH may not be a major contributor to cisplatin resistance in our model. The consequence of ISR-elevated TRB3, HO-1, and PCK2 expression might be related to the cellular stress response rather than to ISR-mediated cisplatin resistance.

xCT (SLC7A11) is a subunit of the xc^-^ system transporter specific for cystine uptake and is essential for intracellular GSH biosynthesis [58,59]. Two amino acid response element (AARE)-like sequences in the promoter region of the SLC7A11 gene provide the ATF4 binding elements to activate the transcription of the SLC7A11 gene [60]. GSH is an important thiol-containing tri-peptide (Glu–Cys–Gly) that provides an important redox buffer in living organisms [61]. Several mechanisms underlying GSH-mediated cisplatin resistance have been proposed. In some cases, GSH may be a cofactor facilitating MRP2-mediated cisplatin efflux [62]. Moreover, GSH may serve as a redox-regulating cytoprotector [63]. In addition, GSH may conjugate with cisplatin to lead to the detoxification of cisplatin. In the present study, we found that salubrinal-induced xCT expression leads to increased GSH biosynthesis and reduced cisplatin-induced lipid peroxidation. Using the xCT inhibitor SSA and the GSH synthesis inhibitor BSO, we demonstrated that inhibition of xCT-mediated GSH biosynthesis may significantly attenuate the salubrinal-induced cisplatin resistance. This finding is consistent with the activation of the eIF2α–ATF4 pathway and the high xCT expression of cisplatin-resistant gastric cancer cells [40]. Our findings suggest that enhanced GSH biosynthesis and reduced cisplatin-induced oxidative stress are responsible for the eIF2α–ATF4–xCT-mediated chemoresistance in gastric cancer cells. This study reveals that ISR is one of the important cisplatin resistance mechanisms through GSH elevation-mediated cytoprotection in multi-target aspects. It was noted that carboplatin is also a common chemotherapy agent for various cancers and is a suitable alternative agent for cisplatin-contraindicated gastric cancer patients. Moreover, GSH has been reported to contribute to carboplatin resistance [64,65]. The ISR-mediated GSH elevation might be a global mechanism for resistance to platinum agents in gastric cancers. This mechanism may need to be verified by an in vivo model.

In the present study, we evaluated the roles of ATF4-regulated TRB3, HO-1, PCK2, and xCT in the ISR-induced cisplatin resistance, which were based on our IPA analysis and our literature review for previous evidence about chemoresistance [40,44,45,46,52,53,55,56,57]. In addition, we noted that some genes (such as CYP1A1, STC2, ASNS, etc.) listed in association with the tumour cell survival, cell proliferation, and cell death also affect the survival of gastric cancer patients under adjuvant chemotherapy (Supplementary Table S3). Their roles in the ISR-induced chemoresistance might need to be further evaluated.

## 4. Materials and Methods

### 4.1. Cell Culture

Human gastric cancer cell lines AGS, AZ521, and NUGC-3 were cultured in RPMI 1640 medium with 10% foetal bovine serum (FBS) and 1% penicillin/streptomycin (P/S). Cells were maintained in a humidified 37 °C incubator with 5% CO_2_. RPMI, FBS, and P/S were obtained from Gibco^TM^ and Thermo Fisher Scientific (Grand Island, NY, USA). The cisplatin-resistant (AGS–CisR) gastric cancer cells were established as reported in a previous study [40]. The cisplatin-resistant gastric cancer cells were maintained in medium containing cisplatin (AGS–CisR: 1 µg/mL). Before the functional assay, the cisplatin-resistant gastric cancer cells were transferred to RPMI 1640 medium without cisplatin for three days. Cisplatin was obtained from Fresenius Kabi oncology (Distt. Solan, H.P., India).

### 4.2. Determination of Cell Viability

Cell viability was analysed by the Sulforhodamine B (SRB) assay. Cells were seeded in a 96-well cell culture cluster at a density of 3–5 × 10^3^ cells per well and cultured for 24 h prior to drug treatment. In the SRB assay, cells were fixed with 10% (*w*/*v*) trichloroacetic acid (TCA) at 4 °C for 1 h and stained with SRB for 30 min at each time point after drug treatment. The excess dye was removed by washing repeatedly with 1% (*v*/*v*) acetic acid. The protein-bound dye was dissolved in 10 mMTris base (Merck Millipore, Billerica, MA, USA) solution for OD determination at 510 nm using a microplate reader. N-acetylcysteine (NAC), hydrogen peroxide (H_2_O_2_), menadione, SRB, TCA, buthioninesulfoximine (BSO), acetic acid, and sulfasalazine (SSA) were purchased from Sigma-Aldrich (St. Louis, MO, USA). Salubrinal was purchased from Tocris Bioscience (Avonmouth, Bristol, UK). 

### 4.3. Annexin V Staining Assay

The apoptosis assay was performed using the Annexin V-fluorescein isocyanate (FITC) Apoptosis Detection Kit (Sigma-Aldrich, St. Louis, MO, USA) according to the manufacturer’s protocol. The Annexin V-FITC fluorescence intensity at the FL1 was determined by flow cytometry (FACS Calibur flow cytometer, Becton Dickinson, Franklin Lakes, NJ, USA). Data were collected and further evaluated using Cell Quest software (Becton Dickinson). 

### 4.4. Detection of the Levels of Intracellular ROS and Mitochondrial ROS

Dichlorodihydrofluorescein diacetate (DCFH-dA) and MitoSOX Red were used to determine the intracellular levels of ROS and mitochondrial ROS, respectively. After incubation with 5 µM DCFH-dA for 30 min or 5 µM MitoSOX Red for 10 min, cells were washed with PBS, trypsinized, and re-suspended in PBS as previously reported [40]. The DCF fluorescence intensity at the FL1 and the MitoSOX Red fluorescence intensity at the FL2 were determined by flow cytometry. The excitation was delivered by a 488 nm argon laser. A minimum of 10,000 cells were collected and analysed. Data were evaluated by Cell Quest software. The relative change in the mean fluorescence intensity was determined. DCFH-dA and MitoSOX Red were purchased from Molecular Probes^TM^, Invitrogen^TM^, and Thermo Fisher Scientific (Eugene, OR, USA).

### 4.5. Kaplan–Meier Plotter Analysis

The Kaplan–Meier (KM) plotter online database (Available online: http://kmplot.com/analysis/) combines the GEO (Affymetrix microarrays only), EGA, and TCGA databases and is operated at the PostgreSQL server. The patient groups were compared by a Kaplan–Meier (KM) survival plot, and the hazard ratios with 95% confidence intervals and log rank *p* values were calculated using online software as previously described [66]. In the present study, the specific genes ATF4 (Affy ID/gene symbol 200779_at), tribbles-related protein 3 (TRB3, Affy ID/gene symbol 218145_at), heme oxygenase 1 (HO-1, Affy ID/gene symbol 203665_at), phosphoenolpyruvate carboxykinase 2 (PCK2, Affy ID/gene symbol 202847_at), and xCT (Affy ID/gene symbol 209921_at) were analysed in the database of gastric cancer patients for KM analysis [66]. In the selected gastric cancer cohort receiving 5-FU based adjuvant therapy, a total of 153 patients were selected and further analysed for overall survival (OS) and progression-free survival (PFS) by KM analysis.

### 4.6. Western Blot Analysis

Cells were lysed by radioimmunoprecipitation assay lysis buffer (RIPA buffer: 50 mMTris-HCl buffer, pH 7.5, containing 0.15 M sodium chloride (NaCl), 0.5% sodium deoxycholate, 0.5% sodium dodecyl sulfate (SDS), 0.1% Triton X-100, 10 µg/mL aprotinin, 2 mMethylenediaminetetraacetic acid (EDTA), 2 mM sodium orthovanadate (Na_3_VO_4_), and 1 mMphenylmethanesulfonyl fluoride (PMSF)). Cell lysate was prepared by collecting the supernatant after centrifugation at 13,000× *g* for 15 min. The protein concentration of the sample buffer was determined using Bradford reagent with bovine serum albumin (BSA) as the standard (Bio-Rad, Hercules, CA, USA). Samples (20 µg) were separated by 8–12% SDS-polyacrylamide gel electrophoresis (PAGE) and transferred onto a polyvinylidene difluoride membrane (PVDF) membrane (Biotrace^TM^, PALL Life sciences, Ann Arbor, MI, USA). The sample membrane was further immunoblotted with primary and secondary antibodies. Signal was developed from the antibody-protein conjugate using a chemiluminescence kit (Immobilon Western Chemiluminescence HRP Substrates, Merck-Millipore, Billerica, MA, USA). Relative band images and intensities were analysed by a luminescence/fluorescence imaging system (GE Healthcare) and multi gauge image analysis software version 3.0 (Fujifilm, Stockholm, Sweden). Aprotinin, PMSF, Na_3_VO_4_, Triton X-100, SDS, polyacrylamide, and α-tubulin antibodies were purchased from Sigma-Aldrich (St. Louis, MO, USA). Tris-HCl buffer and NaCl were purchased from Merck Millipore (Billerica, MA, USA). The ATF4 antibody was purchased from Proteintech Group (Rosemont, IL, USA). Antibodies against p-eIF2α (Ser52) and eIF2α were purchased from Invitrogen^TM^ and Thermo Fisher Scientific (Camarillo, CA, USA). xCT and TRB3 were purchased from Abcam (Cambridge, MA, USA). PCK2 was purchased from Cell Signaling Technology (Beverly, MA, USA). HO-1 was purchased from Enzo Life Sciences (Postfach, Lausen, Switzerland)

### 4.7. Small Interfering RNA (siRNA)-Mediated Genetic Knockdown

Cells were seeded in a 6 cm dish (Corning Inc., Corning, NY, USA) at a density of 4 × 10^5^ cells per well and cultured overnight. The culture medium was replaced with antibiotic-free medium before transfection. Lipofectamine RNAi MAX reagent (Invitrogen^TM^, Thermo Fisher Scientific, Carlsbad, CA, USA) and the indicated concentration of siRNA were dissolved in antibiotic/serum-free RPMI medium. The diluted siRNA was mixed with Lipofectamine for 5 min at room temperature. After the siRNA-lipid complex was formed, the mixture was directly added into antibiotic-free medium and incubated for 48 h for further experiments. The specific ON-TARGET plus^TM^SMARTpool ATF4 (L-005125), solute carrier family 7 member 11 (SLC7A11, xCT, L-007612), TRB3 (L-003754), Hmox1 (HO-1, L-040543), PCK2 (L-006797), and non-target (scramble, D-001810) siRNAs were used in these experiments.

### 4.8. Quantitative Real-Time Reverse Transcription (RT)-Polymerase Chain Reaction (q-RT PCR)

Total RNA was extracted from cultured cells using the TRIzol reagent following the manufacturer’s instructions (Invitrogen, Carlsbad, CA, USA). The RT reaction was carried out on 20 µg RNA using the RevertAid^TM^ reverse transcriptase kit (Thermo Fisher Scientific, Waltham, MA, USA). The cDNA products were further subjected to PCR amplification with KAPA SYBR FAST qPCR Kits (Kapa Biosystems, Wilmington, MA, USA) using the StepOne^TM^ System (Applied Biosystems^TM^ real-time PCR Instrument, Thermo Fisher Scientific). The primer sequences were PCK2, forward: 5′-CAACCAGAGGGCATCCACAT-3′, reverse: 5′-TACTCGTGCCACATCCTTGG-3′; DDIT3, forward: 5′-TCCTGGAAATGAAGAGGAAG-3′, reverse: 5′-TGTGACCTCTGCTGGTTCTG-3′; NUPR1, forward: 5′-AGAAGCTGCTGCCAACACCA-3′, reverse: 5′-TAGTGTCCATGGTCTGGCCTC-3′; xCT, forward: 5′-TCATTGGAGCAGGAATCTTCA-3′, reverse: 5′-TTCAGCATAAGACAAAGCTCCA-3′; PSAT1, forward: 5′-CGGTCCTGGAATACAAGGTG-3′, reverse: 5′-AACCAAGCCCATGACGTAGA-3′; TRB3, forward: 5′-TGGTACCCAGCTCCTCTACG-3′, reverse: 5′-GACAAAGCGACACAGCTTGA-3′; ASNS, forward: 5′-CGACCAAAAGAAGCCTTCAG-3′, reverse: 5′-GCCATCATTGCATCATCAAC-3′; SESN2, forward: 5′-TTCGGATATGAGGACTTCAC-3′, reverse: 5′-ATGGTATTGTAGGTGAGGCT-3′; HO-1, forward: 5′-CAGGCAGAGAATGCTGAGTTC-3′, reverse: 5′-GATCTTGAGCAGGAACGCAGT-3′; and glyceraldehyde 3-phosphate dehydrogenase (GAPDH), forward: 5′-CCGTCTAGAAAAACCTGCC-3′, reverse: 5′-GCCAAATTCGTTGTCATACC-3′. The q-RT PCR was performed by denaturation at 95 °C for 3 min followed by 50 cycles of 95 °C for 3 s and 60 °C for 30 s. Relative gene expression levels were determined by the 2^−∆∆Ct^ method and were normalized to the level of GADPH in each sample.

### 4.9. Microarray Analysis and Ingenuity Pathway Analysis (IPA)

Cellular RNA was extracted from the gastric cancer cells treated with or without 30 µM salubrinal for 24h using the TRIzol reagent following the manufacturer’s instructions (Invitrogen, Carlsbad, CA, USA). RNA samples were hybridized with the Affymetrix Human Genome U133 plus2.0 gene chip (Affymetrix, Santa Clara, CA, USA)), and microarray data were analyzed by the National Yang-Ming University VYM Genome Research Center. The results of microarray results were further analyzed using the gene set enrichment analysis (GSEA) software (version 3.0, Broad Institute, Massachusetts Institute of Technology, and Regents of the University of California) and the ingenuity pathway analysis (IPA) software (QIAGEN, Redwood City, CA, USA).

### 4.10. Glutathione (GSH) Detection

The reduced GSH and total GSH levels were determined using a Glutathione Assay Kit (Sigma-Aldrich, St. Louis, MO, USA) according to the instruction manual.

### 4.11. Lipid Peroxidation Assay

The MDA levels were determined using a lipid peroxidation malondialdehyde (MDA) assay kit (Sigma-Aldrich, St. Louis, MO, USA) according to the instruction manual.

### 4.12. Statistical Analysis

The statistical significance of differences was analysed by Student’s *t* test. A *p*-value < 0.05 was considered to be statistically significant. The data are presented as the mean ± SEM. Sigmaplot software, version 10.0 (Systat Software, San Jose, CA, USA), and GraphPad PRISM software, version 6 (GraphPad Software, La Jolla, CA, USA), were used for the statistical analyses.

## 5. Conclusions

In conclusion, we demonstrated that activation of ISR itself may promote cisplatin resistance through the up-regulation of ATF4-dependent xCT expression, enhanced GSH biosynthesis, and reduced cisplatin-induced oxidative stress. Our findings suggest that the activation of ISR may be a potential drug target for improving the efficacy of gastric cancer treatment.

## Figures and Tables

**Table 1:** The ATF4-regulated genes, tribbles-related protein 3 (TRB3), heme oxygenase 1 (HO-1), phosphoenolpyruvate carboxykinase (PCK2), and xCT, are associated with a poor prognosis in gastric cancer patients after adjuvant chemotherapy.

ATF4-Regulated Genes	Progression Free Survival	Overall Survival
	High vs. Low Expression	High vs. Low Expression
	(Hazard Ratio, HR)	(Hazard Ratio, HR)
TRB3	2.57 (1.69–3.9)	2.55 (1.67–3.89)
	Logrank *p* = 5.3 × 10^−6^	Logrank *p* = 8.9 × 10^−6^
	(*n* = 110 vs. 43)	(*n* = 110 vs. 43)
HO-1	1.55 (1.04–2.3)	1.74 (1.15–2.63)
	Logrank *p* = 0.028	Logrank *p* = 0.008
	(*n* = 111 vs. 42)	(*n* = 111 vs. 42)
PCK2	1.67 (1.15–2.42)	1.84 (1.26–2.69)
	Logrank *p* = 0.0066	Logrank *p* = 0.0013
	(*n* = 102 vs. 51)	(*n* = 43 vs. 110)
xCT	1.43 (1.01–2.02)	1.48 (1.04–2.11)
	Logrank *p* = 0.043	Logrank *p* = 0.027
	(*n* = 71 vs. 82)	(*n* = 82 vs. 71)

## Supplementary Materials

Supplementary materials can be found at http://www.mdpi.com/1422-0067/19/11/3389/s1, Table S1: The gene list of up-regulated expression (over 2-fold) in the results of microarray analysis for 30 µM, 24 h salubrinal treatment, Table S2: The IPA analysis of microarray data for aspects of cell survival, cell proliferation, and cell death, Table S3: Analysis of patient prognosis of the up-regulated gene expression in the gastric tumor specimen from the Kaplan–Meier Plotter dataset.

## Abbreviations

5-FU	5-Fluorouracil
AARE	amino acid response element
ATF4	activating transcription factor-4
ATP7B	copper-transporting ATPase
BSA	bovine serum albumin
BSO	buthioninesulfoximine
CO	carbon monoxide
CTR1	copper transporter 1
EDTA	ethylenediaminetetraacetic acid
eIF2α	eukaryotic translation initiation factor 2α
ERCC1	excision repair cross-complementing rodent repair deficiency, complementation group 1
FBS	foetal bovine serum
GAPDH	glyceraldehyde 3-phosphate dehydrogenase
GCN2	general control nonderepressible 2
GSH	glutathione
H_2_O_2_	hydrogen peroxide
HO-1(HMOX1)	heme oxygenase 1
HRI	heme-regulated eIF2α kinase
IPA	Ingenuity pathway analysis
ISR	Integrated stress response
KM	Kaplan-Meier
MDA	Malondialdehyde
MRP2	multidrug resistance-associated protein 2
mtDNA	mitochondrial DNA
Na_3_VO_4_	sodium orthovanadate
NAC	*N*-acetylcysteine
NaCl	sodium chloride
NADPH	nicotinamide adenine dinucleotide phosphate
OS	overall survival
P/S	penicillin/streptomycin
PAGE	polyacrylamide gel electrophoresis
PCK2	phosphoenolpyruvate carboxykinase 2
PEPCK	phosphoenolpyruvate carboxykinase
PERK	PKR-like endoplasmic reticulum kinase
PFS	progression-free survival
PKR	protein kinase R
PMSF	phenylmethanesulfonyl fluoride
PVDF	polyvinylidene difluoride membrane
q-RTPCR	quantitative real-time reverse transcription-polymerase chain reaction
ROS	reactive oxygen species
RT	reverse transcription
SDS	sodium dodecyl sulfate
SRB	Sulforhodamine B
SSA	sulfasalazine
TCA	trichloroacetic acid
TRB3	tribbles-related protein 3
VRACs	volume-regulated anion channels
