# Supplementary material for: Activated Integrated Stress Response Induced by Salubrinal Promotes Cisplatin Resistance in Human Gastric Cancer Cells via Enhanced xCT Expression and Glutathione Biosynthesis
Source: Int J Mol Sci. 2018 Oct 29;19(11):3389. doi: 10.3390/ijms19113389 (PMC6275069; doi:10.3390/ijms19113389)
Supplement: Supplementary file 1 [file ijms-19-03389-s001.pdf]

International Journal of *Molecular Sciences*

MDPI

Supplementary Table 1. The gene list of up-regulated expression (over 2 folds) in the results of microarray analysis for 30 μM, 24 h salubrinal treatment.

Transcript Cluster ID	Gene Symbol	Fold Change (Sal vs. Con)	Transcript Cluster ID	Gene Symbol	Fold Change (Sal vs. Con)
205749_at	CYP1A1	7.46	226612_at	UBE2QL1	2.28
204971_at	CSTA	6.34	203543_s_at	KLF9	2.21
203438_at	STC2	5.12	202341_s_at	TRIM2	2.16
217678_at	SLC7A11	3.9	205660_at	OASL	2.15
202847_at	PCK2	3.89	220892_s_at	PSAT1	2.14
205047_s_at	ASNS	3.78	1552275_s_at	PXK	2.14
241412_at	BTC	3.18	209383_at	DDIT3	2.13
229354_at	AHRR	3.04	214098_at	KIAA1107	2.12
228640_at	PCDH7	2.79	226614_s_at	FAM167A	2.12
206101_at	ECM2	2.7	235308_at	ZBTB20	2.12
1553249_at	SMC1B	2.63	229778_at	C12orf39	2.11
239468_at	MKX	2.56	241372_at	ZC3H6	2.11
202436_s_at	CYP1B1	2.52	1555912_at	ST7-AS1	2.08
227410_at	FAM43A	2.52	212665_at	TIPARP	2.07
218145_at	TRB3	2.51	230383_x_at	SLFN5	2.07
219270_at	CHAC1	2.47	214748_at	N4BP2L2	2.04
226757_at	IFIT2	2.46	226158_at	KLHL24	2.04
209230_s_at	NUPR1	2.45	219127_at	PRR15L	2.03
232094_at	KATNBL1	2.43	206157_at	PTX3	2.02
210118_s_at	IL1A	2.39	221156_x_at	CCPG1	2.02
226725_at	SLFN5	2.39	222073_at	COL4A3	2.02
205830_at	CLGN	2.37	1557488_at	CBX3P2	2.02
203665_at	HMOX1	2.36	227475_at	FOXQ1	2.01
1553055_a_at	SLFN5	2.33	242088_at	KLHL24	2.01
1555788_a_at	TRB3	2.32	203542_s_at	KLF9	2

Supplementary Table 2. The IPA analysis of microarray data for aspects of cell survival, cell proliferation, and cell death.

Types	Genes	*p*-value
Apoptosis of tumor cells	CSAT, ASNS, CYP1B1, NUPR1, IL1A, HMOX1, KLF9, DDIT3	8.86E-06
Cell death of tumor cells	CSTA, SLC7A11, PCK2, ASNS, CYP1B1, TRB3, NUPR1, IL1A, HMOX1, KLF9, DDIT3	8.21E-07
Cell proliferation	CYP1A1, STC2, CSTA, SLC7A11, PCK2, ASNS, BTC, CYP1B1, TRB3, NUPR1, SLFN5, IL1A, HMOX1, KLF9, DDIT3, ZBTB20, PTX3, COL4A3, CCPG1, FOXQ1	2.42E-06
Cell survival	ASNS, BTC, NUPR1, IL1A, HMOX1, DDIT3, ZBTB20, COL4A3	3.88E-04
Cell viability	ASNS, BTC, NUPR1, IL1A, HMOX1, DDIT3, ZBTB20, COL4A3	1.86E-04
Cell cycle	ASNS, BTC, NUPR1, IL1A, HMOX1, DDIT3, ZBTB20, CCPG1, PTX3, VAV3	3.26E-04

Supplementary Table 3. Analysis of patient prognosis of the up-regulated gene expression in the gastric tumor specimen from the Kaplan-Meier Plotter dataset.

	5-FU based adjuvant OS (N=153)		5FU based adjuvant FP (N=153)	
Genes	P value	HR	P value	HR
CYP1A1	2.60E-04	1.91 (1.34-2.73)	1.40E-04	1.97 (1.38-2.8)
STC2	3.70E-02	1.51 (1.02-2.23)	4.70E-03	1.75 (1.18-2.58)
SLC7A11	2.70E-02	1.48 (1.04-2.11)	4.30E-02	1.43 (1.01-2.02)
PCK2	1.30E-03	1.84 (1.26-2.69)	6.60E-03	1.67 (1.15-2.42)
ASNS	8.10E-04	1.84 (1.28-2.64)	7.20E-04	1.84 (1.29-2.64)
BTC	4.80E-03	0.6 (0.42-0.86)	2.10E-03	0.58 (0.41-0.83)
AHRR	3.90E-02	0.36 (0.13-0.98)	1.70E-02	0.36 (0.15-0.86)
PCDH7*	9.60E-03	299986029 (0-Inf)	1.20E-02	8.58 (1.15-63.95)
ECM2	1.30E-02	0.61 (0.42-0.9)	1.80E-02	0.63 (0.43-0.93)
SMC1B	1.10E-02	0.23 (0.06-0.79)	4.30E-03	0.15 (0.04-0.67)
MKX	3.70E-01	1.75 (0.51-6.03)	5.40E-01	1.3 (0.56-3.03)
CYP1B1	5.40E-06	0.38 (0.25-0.59)	2.20E-06	0.37 (0.24-0.57)
FAM43A	8.80E-02	1.39 (0.95-2.05)	8.60E-02	1.4 (0.95-2.05)
TRB3	8.90E-06	2.55 (1.67-3.89)	5.30E-06	2.57 (1.69-3.9)
CHAC1	4.20E-02	0.67 (0.45-0.99)	5.00E-02	0.68 (0.46-1)
IFIT2	3.70E-02	4.36 (1-19.08)	6.70E-02	2.68 (0.9-8)
NUPR1	3.00E-04	0.5 (0.34-0.73)	1.90E-03	0.56 (0.39-0.81)
IL1A	2.60E-05	2.13 (1.48-3.04)	3.10E-05	2.11 (1.48-3.02)
SLFN5	2.20E-01	0.51 (0.17-1.52)	1.20E-01	0.48 (0.19-1.24)
CLGN	1.80E-01	0.79 (0.56-1.12)	2.40E-01	0.8 (0.54-1.17)
HMOX1	8.00E-03	1.74 (1.15-2.63)	2.80E-02	1.55 (1.04-2.3)
UBE2QL1	2.70E-01	1.77 (0.64-4.95)	4.50E-01	0.68 (0.25-1.87)
KLF9	1.10E-05	0.4 (0.27-0.61)	2.40E-06	0.38 (0.25-0.58)
TRIM2	2.40E-02	0.67 (0.47-0.95)	7.70E-03	0.62 (0.43-0.88)
OASL	7.90E-02	0.74 (0.52-1.04)	2.00E-02	0.67 (0.47-0.94)
PSAT1	3.50E-01	1.54 (0.62-3.81)	3.60E-01	1.47 (0.64-3.36)
PXK	2.20E-02	0.25 (0.07-0.89)	2.70E-02	0.31 (0.1-0.92)
DDIT3	2.20E-01	1.29 (0.86-1.93)	2.50E-01	1.23 (0.86-1.75)

(*: N=34)
